# A minority-group of renal cell cancer patients with high infiltration of CD20+B-cells is associated with poor prognosis

**DOI:** 10.1038/s41416-018-0266-8

**Published:** 2018-10-08

**Authors:** Elin Sjöberg, Magnus Frödin, John Lövrot, Artur Mezheyeuski, Martin Johansson, Ulrika Harmenberg, Lars Egevad, Per Sandström, Arne Östman

**Affiliations:** 1 Department of Oncology-Pathology, Karolinska Institutet, Stockholm, Sweden; 2 Department of Immunology, genetics and pathology, Uppsala University, Uppsala, Sweden; 3 Department of Laboratory Medicine, Lund University, Lund, Sweden

**Keywords:** Tumour biomarkers, Tumour immunology

## Abstract

**Background:**

The role of B-lymphocytes in solid tumours is unclear. Tumour biology studies have implied both anti- and pro-tumoural effects and prognostic studies have mainly linked B-cells to increased survival. This study aimed to analyse the clinical relevance of B-lymphocytes in renal cell cancer (RCC), where information on the prognostic impact is lacking.

**Methods:**

Following immunohistochemistry (IHC) stainings with a CD20 antibody, density of CD20+ B-cells was quantified in an RCC discovery- and validation cohort. Associations of B-cell infiltration, determined by CD20 expression or a B-cell gene-signature, and survival was also analysed in 14 publicly available gene expression datasets of cancer, including the kidney clear cell carcinoma (KIRC) dataset.

**Results:**

IHC analyses of the discovery cohort identified a previously unrecognised subgroup of RCC patients with high infiltration of CD20+ B-cells. The B-cell-high subgroup displayed significantly shorter survival according to uni- and multi-variable analyses. The association between poor prognosis and high density of CD20+ B-cells was confirmed in the validation cohort. Analyses of the KIRC gene expression dataset using the B-cell signature confirmed findings from IHC analyses. Analyses of other gene expression datasets, representing 13 different tumour types, indicated that the poor survival-association of B-cells occurred selectively in RCC.

**Conclusion:**

This exploratory study identifies a previously unrecognised poor-prognosis subset of RCC with high density of CD20-defined B-cells.

## Introduction

Renal cell cancer (RCC) originates from the renal epithelial cells and constitutes ~3 percent of all human malignancies.^1^ Despite advancements in targeted therapies in the last decade, the disease is notoriously difficult to treat once metastasis is manifest, with an 5-year survival rate of only 8–12 percent.^2^ The most common form of RCC is the clear cell type, which make up ~70% of all RCC, and is characterised by an inactivation of the von-Hippel-Lindau gene.^3^ Anti-angiogenic drugs currently used for the treatment of metastatic RCC are based on clinical trials with sunitinib, pazopanib, axitinib and cabozantinib.^4,5^ More recently, immune checkpoint regulators have been added to the armamentarium,^6^ although the mechanism by which they exert their antitumoural function in RCC is not fully understood.

The immune system has during recent years been an attractive target for treatment of several cancers. T-cells are the best characterised immune cells that have an antitumour function and correlate with a favourable prognosis in many tumours.^7,8^ On the contrary, B-lymphocytes are less studied and the role during tumour progression is not fully understood. Also, clinical relevance of these findings remains largely unknown. Some recent studies have reported tumour promoting B-cells in tumours. These tumour promoting functions involve modulation of T-cell-mediated tumour cell killing and responses to chemotherapy^9,10^ or secretion of tumour stimulatory cytokines.^11,12^

Tumour suppressive functions of B-lymphocytes are implied by a number of studies which have identified an association of B-cells and prolonged survival in different cancers, including liver cancer, ovarian cancer, pancreatic adenocarcinoma, melanoma, colorectal cancer, oesophageal cancer, gastric cancer and breast cancer.^13–21^

In some cancers, the association between B-lymphocytes and prognosis is less clear and studies have also reported that B-cells is associated with worse outcome. In patients with primary cutaneous melanoma and ovarian cancer CD20+ and CD138+ B-cells, respectively, correlated to decreased overall survival (OS).^15,22^ In a study by Mahmoud et al., the number of CD20-expressing B-cells in breast tumours was associated with higher tumour grade, hormone receptor negativity and increased breast cancer specific survival.^20^ In a recent study of ductal carcinoma in situ (DCIS), presence of B-lymphocytes also correlated with hormone receptor negativity and large tumour size. However, patients with high B-lymphocyte infiltration had a shorter recurrence free survival.^23^

An extensive analysis of the immune landscape in clear cell RCC was recently published, where a specific immune signature derived from single cell analysis of human tumours correlated to shorter progression free survival.^24^ Associations between B-cell infiltration and survival in RCC have not earlier been reported. In this study we identify a correlation between CD20-defined B-cells, determined by IHC, and worse survival in two independent RCC cohorts. This association was also supported by analyses of publicly available transcriptome data. Furthermore, we show that the association of CD20 expression and poor prognosis appear selective to RCC.

## Material and methods

### Patient material and tissue microarrays

Two RCC tissue microarrays (TMAs) were used in the study.

The discovery cohort is a population-based cohort consisting of 314 RCC patients diagnosed between 1978 and 1996 at Skåne University Hospital Malmö, Sweden. For validation, a second TMA was made from 64 RCC patients diagnosed between 1997 and 2005 at Karolinska University Hospital, Stockholm, Sweden, who all developed metastatic disease which was treated with at least one course of sunitinib as first line treatment. M-stage in this cohort refers to presence of metastasis at diagnosis. Data of patient characteristics, treatment and OS was collected in clinical registries. Details about the cohorts are presented in Table 1. The discovery cohort has been used in previous biomarker studies.^25,26^ For both TMAs tumours were revised by a pathologist and representative tumour parts were chosen. Each tumour was represented by two 1 mm diameter core punch biopsies.

**Table 1:** Associations between CD20-status and clinicopathological parameters in the discovery cohort, validation cohort and KIRC

	Discovery cohort				Validation cohort				KIRC			
	Total (*n*=297)	CD20		*p*-value	Total (*n*=64)	CD20		*p*-value	Total (*n*=534)	*MS4A1/CD19/PAX5*		*p*-value
		Low (*n*=256)	High (*n*=41)			Low (*n*=46)	High (*n*=18)			Low (*n*=477)	High (*n*=57)	
Sex												
Female	127	112	15	0.39^a^	14	10	4	1.0^b^	188	163	25	0.15^a^
Male	170	144	26		50	36	14		345	313	32	
Missing	0	0	0		0	0	0		2	2	0	
Age												
<60	100	86	14	0.98^a^	13	8	5	0.49^b^	265	238	27	0.71^a^
≥60	194	167	27		51	38	13		268	238	30	
Missing	3	3	0		0	0	0		1	1	0	
Histology												
Clear cell	238	206	32	0.37^b^	59	42	17	0.81^a^				
Papillary	11	10	1		3	2	1					
Sarcomatoid	7	7	0		0	0	0					
Chromophobic	3	1	2		1	1	0					
Missing	38	32	6		1	1	0					
T-stage												
1	32	26	6	0.16^b^	10	7	3	0.82^a^	273	255	18	0.002^a*^
2	39	33	6		14	11	3		69	60	9	
3	34	33	1		38	26	12		180	152	28	
4	62	50	12		1	1	0		11	9	2	
Missing	130	114	16		1	1	0		1	1	0	
Fuhrman grade												
1	112	103	9	0.007^b*^								
2	107	92	15									
3	54	39	15									
4	21	19	2									
Missing	3	3	0									
MSKCC												
Low					26	20	6	0.85^a^				
Intermidiate					31	22	9					
High					3	2	1					
Missing					4	2	2					
N-stage												
0									239	215	24	0.084^b^
1									16	12	4	
Missing									279	250	29	
M-stage												
0	239	211	28	0.034^a*^	40	29	11	1.0^b^	421	381	40	0.031^a*^
1	58	45	13		24	17	7		79	65	14	
Missing	0	0	0		0	0	0		34	31	3	

^a^Pearson Chi-square test

^b^Fisher exact test

^*^P-value < 0.05 is considered significant

### Immunohistochemistry staining and evaluation

Tumour sections were deparaffinised and rehydrated in xylene, 99% ethanol, 95% ethanol and 70% ethanol, and then washed in distilled water. Antigen retrieval was obtained in a decloaking chamber (Biocare Medical) at 95 °C for 15 min in pH 9.0 retrieval buffer (Dako, Cat. nr. S2367) followed by cooling in room temperature for 30 min. Endogenous peroxidase was quenched by incubation with 3% H_2_O_2_ (Invitrogen, Stockholm, Sweden) for 10 min. After washing in PBS with 0.1% Tween 20, tumour sections were blocked in Protein Block (Dako, Serum-Free Ready to Use, Cat. nr X0909) for 1 h at room temperature. As primary antibody, CD20 mouse anti-human antibody (Dako, Cat. nr M0755) was used at a concentration of 1:300 and tissue slides were incubated over night at 4 °C. Thereafter, the secondary antibody (HRP-conjugated goat anti-mouse, Dako, Cat. nr. P0447) was added for 1 h at room temperature. For antibody detection, slides were incubated with DAB (Vector Laboratories, Burlingame, CA, USA) for 2 min and counterstaining with haematoxylin (Histolab Products AB, Gothenburg, Sweden) was performed for 30 s, followed by dehydration and mounting with Vectamount permanent mounting media (Vector Laboratories, Inc., Burlingame, CA, USA).

The CD20-stainings were evaluated by two investigators (ES and MF), mentored by board-certified pathologist (LE), and performed blinded with regard to outcome and clinic-pathological characteristics. Each case was assigned a score based on the mean number of CD20+ cells detected in each of the two tissue cores from each case.

### Analyses of gene expression data sets

The association of B-cell infiltration and survival was analysed in a clinical cohort of clear cell RCC with publicly available transcriptome data (KIRC), generated by The Cancer Genome Atlas (TCGA) Research Network (http://cancergenome.nih.gov/). The dataset consists of gene expression data from 534 patients with clear cell RCC. B-cell infiltration was assessed by a B-cell gene expression signature score created by log2-transformation of the Z-score values for *CD19, MS4A1* and *PAX5* in KIRC, obtained from cbioportal. The same cut-off (86-percentile) as for the discovery cohort was used for dichotomisation of patients with low or high B-cell infiltration.

Publicly available gene expression datasets from 14 cancer types from the TCGA database was used to analyse the association between the gene expression of *MS4A1* (CD20) and survival.

### Statistical analyses

For determination of the cut-off value for dichotomisation, The R package flexmix was used to fit a zero-inflated Poisson mixture model of CD20 data in the discovery cohort. The model is a mixture of two Poission distributions (low and high abundance of CD20-positive cells) and a point distribution at zero. A cut-point for dichotomisation into low and high abundance was determined based on the posterior probabilities.^27^

Association of CD20 + staining or the B-cell signature with clinic-pathological parameters was analysed with Fisher exact test or Pearson Chi-square test.

The duration of survival time was calculated from the date of diagnosis to the date of death or last known follow-up. Probabilities of survival were estimated using the Kaplan–Meier method and log-rank test. The correlation of CD20 status with outcome was evaluated using Cox proportional hazards regression model in uni- and multi-variable analyses.

Statistical analyses were done using the SPSS software package 21.0 (IBM Corporation, Armonk, NY). *P*-values <0.05 were considered statistically significant.

## Results

### A minority-group of RCC patients display high infiltration of CD20+ B-cells

Exploratory analyses were performed to characterise inter-case variations with regard to infiltration of B-cells, as determined by IHC staining of CD20, in a population-based cohort of RCC (referred to as the discovery cohort).

**Fig. 1:**
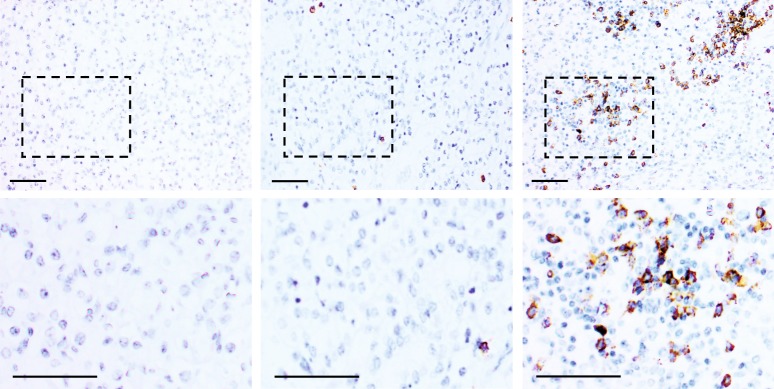
Variable infiltration of CD20+ cells in RCC. Immunohistochemistry staining with CD20 antibodies was used to identify tumour infiltrating B-lymphocytes in RCC patients. The majority of patients displayed no (left panel) or low infiltration (middle panel), whereas a minority of cases demonstrated high infiltration of CD20+ cells (right panel). Scale bar 100 μm

Of the 314 RCC patients included in the TMA, 297 yielded informative staining. Of these 297 patients, the large majority displayed no infiltration or low infiltration of CD20+ B-lymphocytes (Fig. 1, left and middle panel). However, a minority group of patients was detected with prominent infiltration of CD20+ cells (Fig. 1, right panel). Statistical analysis of the distribution of CD20+ B-cells among patients established a cut-off of 16 cells/core for division of patients into two groups (Supp. Figure 1 and Material and Methods for details). This cut-off generated a subgroup of patients with high B-cell infiltration composed of 14% of the population.

Initial analyses to explore the clinical relevance demonstrated that the B-cell-high subgroup was significantly associated with high Fuhrman grade (*p*-value = 0.007) and metastasis (*p*-value = 0.034), but no associations were detected between B-cell-status and sex, age, tumour stage or histology (Table 1).

Together these analyses identify a previously unrecognised subset of RCC patients with high infiltration of CD20 + B-cells.

### High infiltration of B-cells is an independent poor prognosis marker in renal cancer

The data on tumour infiltrating CD20+ B-cells were combined with patient survival data to explore the potential correlation between CD20-status and outcome.

**Fig. 2:**
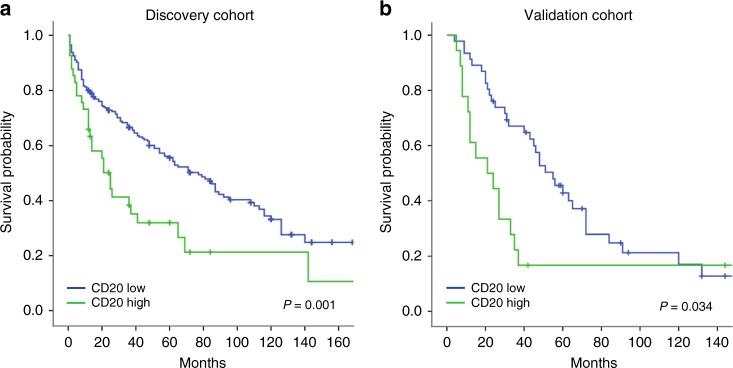
Correlation of CD20 status and prognosis of RCC patients in two independent cohorts. Kaplan–Meier curves showing the overall survival of RCC patients with low or high infiltration of CD20+ cells in the discovery cohort (left panel) and the validation cohort (right panel)

As shown in Fig. 2, Kaplan–Meier analysis demonstrated that the B-cell-high group displayed significantly shorter OS (*p*-value < 0.001; Log-rank-test) (Fig. 2a). Uni-variable Cox-regression analysis confirmed these findings and revealed and increased risk of cancer related death for patients with high infiltration of B-cells (HR = 1.98; 95% CI = 1.32–2.96; *p*-value < 0.001).

Furthermore, multi-variable analyses, also including sex, age, histology, T-stage, M-stage and Fuhrman-grade, demonstrated independent prognostic significance of B-cell status in RCC patients (HR = 2.7; 95% CI = 1.3–5.6; *p*-value = 0.008) (Table 2).

**Table 2:** Multivariable analysis for overall survival in the discovery cohort

	HR	95% CI	*p*-value
Sex			
Female	1		
Male	0.8	0.4–1.3	0.32
Age			
<60	1		
≥60	2.6	1.4–4.8	0.002^*^
Histology			
Non clear cell	1		
Clear cell	0.2	0.1–0.5	<0.001^*^
T-stage			
1	1		
2	1.4	0.4–5.1	0.65
3	1.8	0.5–7.1	0.38
4	3.9	1.1–13.9	0.03^*^
Fuhrman grade			
1	1		
2	1.6	0.8–3-1	0.2
3	1.6	0.7–3.3	0.2
4	3.5	1.3–9.2	0.01^*^
M-stage			
0	1		
1	5.7	3.1–10.7	<0.001^*^
CD20+ B-cells			
Low	1		
High	2.7	1.3–5.6	0.008^*^

^*^P-value < 0.05 is considered significant

To validate these findings, CD20-status was determined in an independent cohort of RCC (referred to as the validation cohort) composed of patients, which at metastatic stage had been treated with sunitinib (see Material and Methods for details). As in the population-based discovery cohort a minority B-cell-high group (28%) was identified also in this cohort, using the same cut-off (16 cells/core). In this cohort, B-cell-status was not significantly associated with any clinic-pathological parameters including sex, age, histology, MSKCC-grade, T- or M-stage (Table 1).

Survival analyses in the validation cohort confirmed a significant association between shorter OS and B-cell-high status in RCC patients as shown in Fig. 2b (*p*-value = 0.034; Log-Rank-test), and an increased risk of death in the CD20-high group as determined by uni-variable Cox Regression analysis (HR = 1.9; 95% CI = 1.0–3.6; *p*-value = 0.039). Furthermore, CD20-status demonstrated independent prognostic significance in multi-variable analyses including clinico-pathological characteristics (HR 2.9; 95% CI = 1.4 – 6.4; *p*-value = 0.005) (Table 3).

**Table 3:** Multivariable analysis for overall survival in the validation cohort and KIRC

Validation cohort				KIRC			
	HR	95% CI	p-value		HR	95% CI	p-value
Sex				Sex			
Female	1			Female	1		
Male	0.9	0.4–2.2	0.9	Male	1.2	0.8–1.8	0.5
Age				Age			
<60	1			<60	1		
≥60	0.5	0.2–1.2	0.1	≥60	2.0	1.3–3.3	0.002^*^
Histology							
Non clear cell	1						
Clear cell	2.1	0.6–7.9	0.3				
T-stage				T-stage			
1	1			1	1		
2	1	0.3–3.0	0.9	2	0.8	0.4–1.7	0.5
3	2.7	1.0–7.1	0.04^*^	3	2.0	1.2–3.4	0.007^*^
4	0.9	0.1–8.8	0.9	4	2.2	0.6–7.9	0.2
MSKCC				N-stage			
Low	1			0	1		
Intermediate	2.9	0.8-3-1	0.05^*^	1	2.5	0.9–6.3	0.06
High	5.3	0.7–3.3	0.021^*^				
M-stage				M-stage			
0	1			0	1		
1	1.9	0.9–4.1	0.1	1	3.3	1.9–5.5	0.001^*^
CD20+B-cells				MS4A1/CD19/PAX5			
Low	1			Low	1		
High	2.9	1.4–6.4	0.005^*^	High	0.6	0.31–1.3	0.2

^*^P-value < 0.05 is considered significant

Together these analyses, using two different patient cohorts, identify infiltration of CD20 + B-cells as a novel independent marker for RCC survival.

### A B-cell signature is correlated with poor prognosis in the TCGA clear cell RCC cohort

To extend the IHC-based analyses, the clinical impact of a B-cell infiltration was analysed in the TCGA gene expression dataset of clear cell RCC (KIRC). B-lymphocytes express additional markers besides CD20. Other well-established markers for B-cells are CD19 and the B-cell transcription factor PAX5.^28^

We therefore composed a B-cell gene signature composed of *MS4A1* (CD20), *CD19* and *PAX5*, and a signature score was established for each patient. Based on the dichotomisation of the discovery cohort described above, a patient group of high B-cell signature score was defined composed of the 14% of cases with highest score. Initial analyses demonstrated that this subgroup of patients with high B-cell signature displayed significant positive association with tumour stage (*p*-value = 0.002) and metastasis (*p*-value = 0.031) (Table 1).

**Fig. 3:**
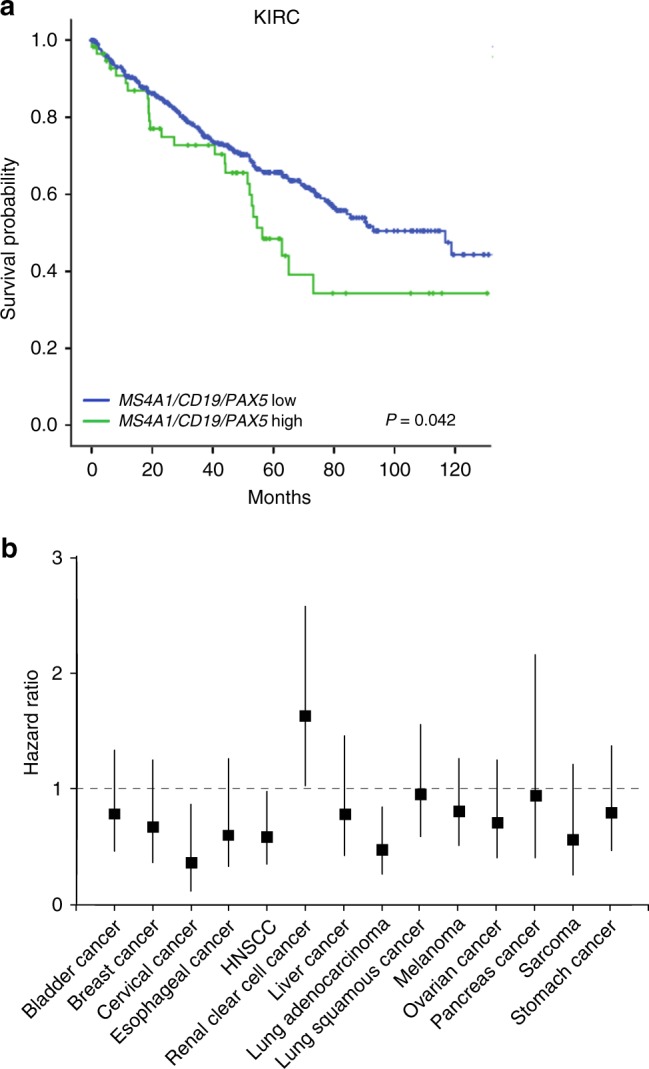
Associations between survival and a B-lymphocyte signature or *MS4A1* expression in gene expression datasets of different tumour types. **a** Kaplan–Meier plot showing overall survival of clear cell RCC patients in the KIRC gene expression dataset (TCGA) with low or high B-lymphocyte gene signature score (*MS4A1/CD19/PAX5*). **b** Association of *MS4A1* expression and overall survival in 14 cancer gene expression datasets from the TCGA database

Survival analyses demonstrated a significantly shorter OS in the B-cell-signature-high group as determined by Log-Rank-test (Fig. 3a; *p*-value = 0.042) and uni-variable Cox-regression analyses (HR = 1.54; CI = 1.01–2.33; *p*-value = 0.043). The B-cell-signature did not remain significant in multivariable analysis including sex, age, T-, N- and M-stage (Table 3).

This signature-based analysis thus supports findings from the IHC analyses indicating the existence of a minority-group of RCC with high B-cell-infiltration and poor prognosis.

### *MS4A1*-expression is not associated with poor prognosis in other common types of cancer

The general understanding that B-lymphocytes are part of an anti-tumour immune response has during recent years been challenged. However, prognostic studies supporting a clinical relevant tumour promoting effect of B-cells is limited.

We therefore performed screening-like exploratory analyses of potential survival associations of high B-cell infiltration, defined by high expression of the gene for CD20 (*MS4A1*), in other tumour types through analyses of a selection of 14 publicly available datasets (including KIRC). For these analyses the minority B-cell-high groups were defined as the 10% of cases with highest *MS4A1* expression.

In agreement with previous findings, the *MS4A1*-high group in RCC showed a significant association with poor survival (HR = 1.63; CI = 1.03–2.59; p-value = 0.039) (Fig. 3b). In most cohorts, no significant associations were detected between *MS4A1-*status and survival (Fig. 3b). Notably, high *MS4A1*expression was associated with good prognosis in cervical cancer, head and neck squamous cell carcinoma (HNSCC) and lung adenocarcinoma (Fig. 3b).

Collectively, these studies thus indicate that B-cells are associated with poor prognosis selectively in RCC.

## Discussion

This exploratory study of two independent RCC collections identifies a previously unrecognised minority-subset of RCC defined by high infiltration of CD20+ B-cells, which is associated with poor prognosis. The existence of this subset is further supported by analyses of the TCGA clear cell RCC gene expression dataset, which confirmed an association between poor prognosis and high expression of either the gene for CD20 or a three-gene B-cell signature. Moreover, the poor prognoses signal of CD20-expressing B-cells was exclusively found in RCC. The cases of the large discovery cohort of the present study did not receive any anti-angiogenic drugs. The survival associations of this study are thus likely reflecting aspects of the natural course biology of RCC.

These correlative studies suggest the possibility of a subset of RCC where B-cells exert pro-tumoural functions. Model-based studies have suggested numerous mechanisms whereby B-cells can stimulate tumour growth and alter response to therapy. These include production of autoantibodies, complement conjugation and secretion of immune-regulatory cytokines that affect macrophage and T-cell responses.^12^

In a mouse model of squamous carcinoma, CD20+ B-lymphocytes affect tumour growth and decrease response to chemotherapy by altering a macrophage dependent T-cell response.^9^ In line with this, targeting of B-cells in a mouse model of pancreatic cancer modulated macrophage function, restored tumour killing by T-cells and improved the response to chemotherapy.^10^

Some of the tumour promoting effects has been assigned to specific B-cell subsets. A recent study on pancreatic ductal adenocarcinoma identified a B-cell subpopulation that supported early tumour growth by secretion of IL-35.^11^ Additional tumour stimulatory subsets of B-lymphocytes characterised by PD1 expression or CXCL13 secretion have also been identified in mice.^29,30^

CD20 and CD19 are considered pan-markers for B-cells.^12^ Future analyses of the marker- and gene expression status of the B-cells in the poor-prognosis subset of RCC should therefore give some guidance about the possible relevance of the above described mechanisms for the observed survival-associations in this study. Continued characterisation of B-cell subpopulations across tumour types might also help understanding the mechanisms behind the observed selective poor-prognosis association of CD20+ B-lymphocytes in RCC.

The present analyses have not addressed to what extent the B-cell-high subgroup is strongly correlated to specific genetic or epigenetic changes, to certain gene expression signatures or to overall mutation load. Further analyses of the TCGA RCC dataset should be productive in this regard. Possibly such studies can also shed light on the presently unexplained RCC-selectivity of the CD20-high survival association.

Immunotherapy with T-cell modulating antibodies is emerging as a new treatment option in RCC.^31^ Future work should consider the possibility that the B-cell-high RCC-subgroup displays particular response patterns to these treatments. Similarly, possible relationships to sensitivity to anti-angiogenic treatments should also be explored. Future studies on well-matched groups, ideally derived from clinical trials, with or without anti-angiogenic or immunotherapy treatment are therefore warranted to explore relationships between the B-cell marker and response to treatment. Other important tasks for future validation studies include refinement of scoring criteria and identification of optimal cut-offs for dichotomisation. Towards potential clinical utility of the marker, these efforts should aim for a combination of prognostic effect and high feasibility.

One notable implication of the observation that CD20+ B-lymphocytes is associated with worse survival of RCC patients, is that different CD20-directed therapies, presently approved for B-cell lymphomas^32^ can be considered as a treatment regimen for this novel subset of RCC. In this context novel mouse models of RCC should be useful tools for pre-clinical studies.^3^

In summary, findings from this exploratory study suggest future studies to be performed on larger clinically, genetically and molecularly well-annotated RCC cohorts. Such studies should provide additional information about the possibility to develop CD20-status to a biomarker of clinical utility for RCC. Equally important, such studies should also give further information on molecular and genetic features that are associated with CD20, and suggest mechanistic relationships that can have therapeutic implications.

## Electronic supplementary material

Supplementary Figure 1

## Availability of data and material

The data and materials from the study are available upon request.
