# Supplementary material for: A minority-group of renal cell cancer patients with high infiltration of CD20+B-cells is associated with poor prognosis
Source: Br J Cancer. 2018 Oct 8;119(7):840–6. doi: 10.1038/s41416-018-0266-8 (PMC6189087; doi:10.1038/s41416-018-0266-8)
Supplement: Supplementary file 1 — Supplementary Figure 1 [file 41416_2018_266_MOESM1_ESM.docx]

Supplementary Figure 1


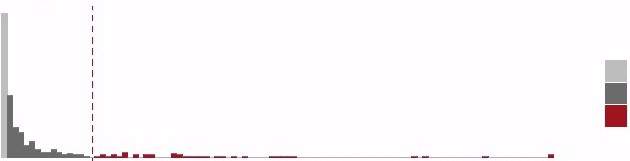
Discovery cohort

90

Nr of patients

60

30

0

0 25 50 75 100

Nr of CD20+ cells

Zero Low High

**Supplementary Figure 1: Distribution of cases according to number CD20+ cells.** Histograms showing number of cases in the discovery cohort with indicated number of infiltrating CD20+ cells.
